# Effects of Growth Medium and Water Stress on Soybean [*Glycine max* (L.) Merr.] Growth, Soil Water Extraction and Rooting Profiles by Depth in 1-m Rooting Columns

**DOI:** 10.3389/fpls.2020.00487

**Published:** 2020-04-24

**Authors:** Michael Gebretsadik Gebre, Hugh James Earl

**Affiliations:** Department of Plant Agriculture, University of Guelph, Guelph, ON, Canada

**Keywords:** volumetric soil water content, soil water profile, rooting profile, dry matter, water stress, controlled environment phenotyping, abiotic stress tolerance, soybean

## Abstract

The pattern of soil water availability in frequently watered small pots is different from field environments. In small pots, volumetric soil water content (VSWC) is relatively high throughout the rooting zone due to a lack of suction to remove water from large and midsize capillaries. This necessitates the use of growing media with large pore space to avoid anaerobic conditions and so prohibits the use of field soil (FS) in small pots. We hypothesized that in 1-m rooting columns, the 0.01-MPa gravitational potential difference between top and bottom may permit the use of lightly-amended FS as a growing medium and provide for realistic VSWC and rooting profiles by depth. This study aimed to investigate the effects of amending a typical sand-based potting mix with different proportions of FS on soybean growth [dry matter (DM) accumulation], water use, VSWC and rooting profiles by depth under control and water stress conditions, in 1-m rooting columns (polyvinyl chloride tubes having an inside diameter of 10 cm and length of 1 m). We tested three growth media (0, 50, and 67% FS mixes), watered daily to either 100% of the maximum soil water holding capacity (SWHC; control) or 75% SWHC (stress). VSWC was calculated from time-domain reflectometry measurements. Compared to all growth media, the 67% FS mix resulted in the highest DM accumulation, water use, water use efficiency (WUE), and also produced realistic VSWC and rooting profiles by depth similar to those reported in the literature under field conditions. Compared to the control, the water stress treatment reduced shoot DM by 24%, root DM by 13%, whole-plant DM by 22%, and water use by 25%, but increased root-to-shoot DM ratio by 18% and WUE by 6%. Of the three growth media tested, the 67% FS mix was the most suitable growth medium for controlled environment phenotyping studies of root functional traits affecting drought tolerance in soybean. This study provides novel phenotyping tools to select for root function and yield formation traits that could increase soybean yield under soil water deficit conditions.

## Introduction

Soybean [*Glycine max* (L.) Merr.] is the number one field crop grown in ON, Canada, with a cultivated area of more than 1.3 million ha and a value of over $1.7 billion in 2017 (OMAFRA, 2019). It is grown mostly under rainfed conditions, so soil water deficits occurring during critical stages of crop development significantly limit Ontario’s soybean yield in most growing seasons, with demonstrated losses in field experiments ranging from 8 to 24% (Hufstetler et al., 2007; Earl, 2012). Even in unusually wet years, soybean yields in Ontario are reduced by transient soil water deficits, and in drier years, yield losses may exceed 25% (H. J. Earl, unpublished data).

Most plant phenotyping experiments that are conducted in controlled environments use small pots (less than 30 cm tall) and commercial potting mixes. In small pot experiments, the pattern of plant water use in response to soil drying is very well studied in soybean and other crop species. Such experiments uniformly show that plants maximize their water use until the soil dries to some critical threshold, below which plant water use and photosynthesis decline linearly (Townend and Dickinson, 1995; Ray and Sinclair, 1997, 1998; Hufstetler et al., 2007; Jones, 2007; Pang et al., 2017; Sinclair et al., 2017). There is genetic variation in soybean for the critical soil water content at which water use begins to decline (Hufstetler et al., 2007), indicating that different genotypes make differing “decisions” about how to respond to reduced water availability.

A limitation of all such experiments is that the pattern of soil water availability in frequently watered small pots used in most plant-water relationship studies is very different from what is encountered in a field environment. For example, a soil in a small pot holds much more water per unit volume than the same soil in the field. This is because, in a pot, small and mid-sized capillary pores tend to be completely filled rather than being drained by suction from lower soil depths (Earl, 2002, 2003; Passioura, 2006; Gebre and Earl, unpublished data) since the gravitational soil water suction (negative pressure) in the field is between 10 and 30 kPa (Chard and Bugbee, 2005; Passioura, 2006) compared to ∼2 kPa gravitational suction in small pots. Secondly and more obviously, in a small pot the root system of the plant explores the entire pot volume very rapidly so that rooting traits such as rate of root elongation or final rooting depth have almost no effect on the plant’s ability to access soil water. Third, plants in small pots require frequent watering since the amount of available water is small relative to daily plant transpiration; this frequent watering from the top required to avoid water stress in small pots tends to prevent the development of soil water gradients with depth that typically occur in a field environment.

While carefully designed small pot experiments can be useful for measuring certain types of plant responses to soil water deficits, in general they provide little useful information about the role of root growth and root function in these responses. Accordingly, the rooting environment in small pots is not a realistic simulation of the field rooting environment. Therefore, to alleviate those problems associated with small pots and permit a meaningful study of root distribution by depth and soil water profiles under controlled environment conditions, we wanted to develop a culture system that could better emulate field conditions.

The relationship between VSWC and soil matric potential is known as the soil water characteristic curve (SWCC). This relationship can be modeled across a broad range of matric potentials (e.g., 0 to 100 MPa) with empirical functions such as the van Genuchten curve (van Genuchten, 1980). In 1-m rooting columns such as those used in the present work, the soil water profile (change in VSWC with height above the bottom of the column) when VSWC is equilibrated against free drainage, is governed by the shape of this relationship in the range of 0 to 0.01 MPa. For example, at the top of the soil column water drains until the positive gravitational potential (0.01 MPa) is in equilibrium with the negative matric potential. It is well established that the shape of the SWCC in this range of matric potentials is a strong function of soil texture (e.g., surface area to volume ratio because of particle sizes), with sandier soils experiencing a much larger decline in VSWC with a given change in matric potential than do finer-textured soils (Fredlund et al., 2002; Tuller et al., 2004; Chard and Bugbee, 2005). In other words, when watered to equilibrium against drainage, VSWC will decline more quickly with height in a column of sandy soil, than in a column of mid-textured soil; soils with more clay and less sand have smaller particles so more surface area to adhere water and thus more VSWC at any given water potential.

Sand-based mixes in small (25 cm tall) pots ensure adequate air space to avoid anaerobic conditions after watering to free drainage. However, preliminary experiments in our lab demonstrated that such potting mixes (for example, the growth medium treatment labeled as 0% FS in the current study) are not suitable for use in 1-m rooting columns. This is because the 0.01 MPa gravitational suction in a 1-m rooting column makes a sand-based mix too dry at the top part of the soil profile to support plant growth (e.g., Figures 5, 6 from the present study; H. J. Earl, unpublished data).

We hypothesized that in 1-m rooting columns, the increased gravitational potential difference between the top and bottom of the soil profile may permit the use of lightly-amended FS as a growing medium and, even at maximum SWHC, provide adequate air space in the top part of the profile to prevent anaerobic conditions. The less extreme soil water release curve (change in VSWC with depth at 100% SWHC) of such a soil compared to a sand-based mix could also produce a more uniform soil water profile over the entire 1 m depth, better emulating what is typically observed under field conditions.

In the present study, we tested three growth media with 0, 50, and 67% FS mixes, watered daily to either 100% (control) or 75% (water stress) of maximum SWHC in 1-m rooting columns. We were not able to include a 100% FS mix as a treatment in this study because in preliminary experiments the mid-textured soil we were using (see section “Materials and Methods”) displayed soil crusting and severe slumping (up to 30 cm) over the course of the experiment.

We predicted that soil mixtures (growth media) with higher ratios of field soil (i.e., the 50 and 67% FS amended mixes) would have a greater SWHC since water is held more tightly in the micro pores of the field soil, and also a more uniform soil water profile from top to bottom since sandy soils generally have a larger change in VSWC between 0.00 and 0.01 MPa matric potential. Relative to the same soil in small pots, at saturation the 1-m rooting columns would have lower VSWC in the upper soil layers, which would eliminate hypoxic conditions normally experienced by soybean grown in field soil mixtures. We expected that watering the rooting columns to 75% rather than 100% SWHC would benefit plant growth in the heavier textured soil mix (67% FS mix) but would be detrimental to plant growth in the soil mix with no field soil added to it (0% FS mix). We also predicted that, contrary to the situation in small pots where roots tend to be quite evenly distributed throughout the entire pot volume, in 1-m rooting columns there would be a more field-like root biomass distribution, with the majority of roots in the upper layers of the soil profile.

Our overall goal of this research was to provide new phenotyping tools for soybean breeders to select root traits that would improve soybean drought tolerance and yield under soil water deficit conditions. The specific objective of this particular study was to explore the effects of amending a typical sand-based potting mix with different amounts of FS on soybean growth [dry matter (DM) accumulation], water use, soil water extraction and rooting profiles by depth under control and water stress conditions, in 1-m rooting columns.

## Materials and Methods

### Construction of Rooting Columns (Tubes)

**FIGURE 1:**
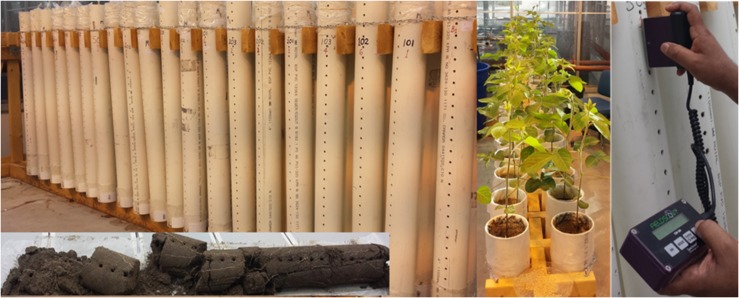
Culture system developed for studying rooting traits in soybean using 1-m rooting columns (tubes). Tubes are drilled on the sides to allow for time-domain reflectometry measurements of volumetric soil water content. Plastic liners allow for the removal of intact root systems, so that root distribution can be accurately determined by depth.

The rooting columns, hereafter referred to as tubes, were made of polyvinyl chloride (PVC; ONYX Hose and Tube Inc., Guelph, ON, Canada), having an inside diameter of 10 cm. PVC tube was purchased as 6 m long units and cut to lengths of 100 cm. Holes, having an inside diameter of 0.6 cm, were drilled down the side of the PVC tubes every 3 cm to allow time-domain reflectometry (TDR; Field Scout^TM^ TDR100 Soil Moisture Meter, Spectrum Technologies, Inc., Aurora, IL, United States) measurements of volumetric soil water content (VSWC; Figure 1). The length of the TDR rods was 3 inches (7.62 cm). A plastic liner measuring 110 cm in length, 2 mils (0.0508 mm) in thickness, and with an inside diameter of 10 cm (Home Depot, Guelph, ON, Canada) was then placed inside each tube to aid in the extraction of the intact soil column and soybean root system at the time of harvest.

A PVC end cap (ONYX Hose and Tube Inc., Guelph, ON, Canada) having an inside diameter of 10.8 cm and a height of 4.5 cm was prepared by drilling a hole (0.8 cm diameter) through the center of the base for drainage. A rectangular piece was removed, 4 cm from the top and 2 cm wide, to aid in taking TDR readings through the bottom TDR access hole. A circular piece of 1 mm mesh nylon screen was fitted to the inside of the end cap to prevent soil from being washed out of the bottom of the tubes through the drainage hole. Each tube was then fitted with the prepared PVC end cap oriented so the bottom TDR hole was exposed through the rectangular gap. A black strip of “Gorilla Duct Tape” (Uline Canada, Milton, ON, Canada), 4.8 cm wide and 1 m long, was used to seal the side holes of the tubes at all times except when TDR measurements were taken. The weight of each tube (including plastic liner, end cap, mesh screen, and duct tape) was recorded.

### Preparation and Potting of Growth Media

For this study, a FS classified as a “*London loam*” was collected from the upper 15 cm at the Elora Research Station (Elora, ON, Canada). The FS was a silty loam (silt = 50%, sand = 31%, clay = 19%) texture that contained 4.2% organic matter, 22.5 ppm P, 61.5 ppm K, 280 ppm Mg, 2375 ppm Ca, 15 ppm Na, 14.4 meq 100 g^–1^ CEC, and had a pH of 7.4 according to a soil test performed by A&L Canada Laboratories Inc., London, ON, Canada.

When preparing the three growth media for this study, the FS was amended with different amounts of granitic sand (B-sand; Hutcheson Sand and Gravel Ltd., Huntsville, ON, Canada) and peat-based potting mix (PGX; Premier Tech, Brantford, ON, Canada). The first soil mix consisted of PGX and granitic sand in a 1:2 v:v ratio (0% FS mix; a mixture of 1/3 PGX and 2/3 granitic sand by volume). This soil mix has been used in many experiments in the past to successfully grow soybean plants in short (25-cm) pots (H. J. Earl, unpublished data). The second soil mix was PGX, granitic sand and FS in a 1:2:3 v:v:v ratio (50% FS mix), and the third soil mix was in a 1:2:6 v:v:v ratio (67% FS mix). Before preparing the 50% FS and 67% FS soil mixtures, the FS was sieved to remove large aggregates to obtain a soil mix with a consistent density and structure. The three soil mixtures had a similar bulk density (∼1.2 g cm^–3^) and total porosity (∼55%). TDR calibration curves were developed for each soil mix across VSWC determined gravimetrically (Supplementary Figure 1).

The soil mix loaded into each tube contained a commercial 20-20-20 N-P-K plus micronutrients fertilizer (Master Plant Products Inc., Brampton, ON, Canada) at the rate of 0.8 g tube^–1^ dissolved in 100 mL water. This fertilizer solution was thoroughly mixed with the soil mixtures before they were loaded into the tubes. This was done so that all soil mixtures were slightly wetted with the fertilizer solution to overcome hydrophobicity of the dry soil and so that the fertilizer was evenly applied throughout the soil depth. During the process of potting, the tubes were filled in a systematic fashion of loading and packing until the soil reached approximately 1 cm below the top part of the tube. The total weight of each tube with its soil was then recorded.

### Determining Soil Water Holding Capacity

To determine the soil water content and mass of dry soil in each tube, samples of each soil mix were taken during the potting process and dried in a forced-air drier at 80°C until a constant weight was attained. All the tubes in all treatments were then watered until they started dripping water from the drainage holes. After 24 h, they were watered again to free drainage to ensure that the soil was completely saturated. Elastic bands were used to close the plastic liners at the tops of the tubes to prevent surface evaporation. The tubes were allowed to drain until a constant weight was achieved (tube weight at the maximum SWHC). The weight of the dry soil and the tubes (tube + liner + cap + screen + duct tape) were then subtracted from this weight to determine the soil water content at maximum SWHC. Then, the target weight for each tube was calculated as the tube + soil dry weight, plus the water weight at maximum SWHC measured for that tube multiplied by the target fraction of maximum SWHC (either 100 or 75%, depending on the treatment).

### Plant Material and Growth Conditions

**FIGURE 2:**
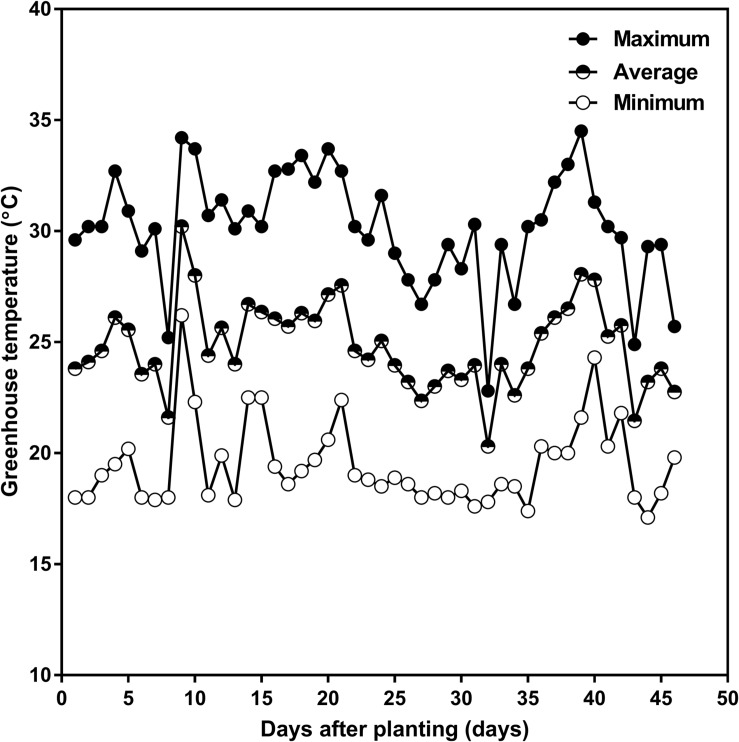
Daily minimum (open circle), average (half-closed circle), and maximum (closed circle) greenhouse air temperature as a function of days after planting in the 2015 summer season, in the greenhouse attached to the Crop Science Building at the University of Guelph. The planting date was July 10 and the harvest date was August 24, 2015.

Plants were grown in the Crop Science Building’s greenhouse at the University of Guelph (43.5314° N, −80.2244° W), Guelph, ON, Canada, in the 2015 summer season. A single Ontario-adapted commercial soybean variety *OAC Bayfield* was sown on July 10 four seeds per tube at 3 cm depth, and then thinned after emergence to one per tube. Greenhouse target temperatures were set at 25°C during the day and 20°C during the night with an average relative humidity of 80%. The actual greenhouse daily minimum, maximum, and average temperatures are given in Figure 2. Natural sunlight was supplemented with overhead high-pressure sodium and metal halide lamps to provide a supplementary 400 μmol m^–2^ s^–1^ photosynthetic photon flux density (PPFD) at the top of the canopy during the photoperiod, and to provide daylength extension to achieve 16 h of light and 8 h of dark. Prior to thinning, tubes were lightly watered daily to prevent soil-crust formation and to keep the soil moisture as uniform as possible. After seedling establishment and thinning, at the V1 (one unrolled trifoliate leaf) developmental stage, the soil water content in each tube was returned to 100% of the maximum SWHC daily by weighing and watering until the V2 (two unrolled trifoliate leaves) developmental stage.

### Experimental Design, Stress Treatments, and Measurements

The experiment was arranged as a 2 × 3 factorial randomized complete block design, with two watering (watered daily to either 100 or 75% of the maximum SWHC) and three soil mix (0, 50, or 67% FS mixes) treatments, replicated six times. The 36 experimental units tubes were placed on a custom-designed wooden stand (Figure 1), arranged in two rows. Four tubes (two at each end) were used to grow border plants to minimize end effects.

Plant growth and development parameters were collected once per week for the duration of the study, beginning 5 days after planting. All tubes were weighed and watered daily to maximum SWHC until the V2 developmental stage (15 days after planting; DAP). At this developmental stage, watering treatments were imposed and lasted through the R3 (beginning pod) developmental stage. During this period (V2 to R3 stages), plants were watered according to the treatment: tubes were returned to either 100% (control) or 75% (water stress) of the maximum SWHC by daily weighing and watering. Total plant water use per day was calculated from the water additions. The whole-plant water use from planting to harvest was calculated as water use (g plant^–1^) = [total amount of water added to each tube from planting to harvest + (starting weight−end weight of each tube at harvest) + whole-plant fresh biomass at harvest].

Time-domain reflectometry millisecond readings were recorded twice a week for the duration of the study, beginning right at the planting date. Before every set of TDR measurements, the TDR meter calibration procedure was performed. The TDR measurements were performed from the top part of the soil profile every 3 cm down to the bottom part of the soil profile via the pre-drilled holes in the sides of the tubes. The TDR measurements were always made just before daily watering (i.e., 24 h after the previous watering), except on two dates when TDR was measured 6, 24, and 48 h after watering to monitor the pattern of soil water depletion over time (Figure 7 and Supplementary Figure 4). The VSWC (%) was calculated from the TDR millisecond readings for each soil mix using the calibration curves developed for that soil mix (Supplementary Figure 1). At harvesting date (46 DAP), plant height and SPAD readings were measured. SPAD readings for chlorophyll content analysis were carried out on the central leaflet of the top fully expanded trifoliate leaf, non-destructively using a Minolta SPAD 502 Plus Chlorophyll Meter (Spectrum Technologies Inc., Aurora, IL, United States).

### Harvest and Postharvest Procedures

At the end of the experiment (46 DAP), all plants were cut at soil level and aboveground plant fresh biomass data was recorded immediately after harvest, and samples were placed in a labeled paper bag for oven drying. The soil and the intact root systems within each rooting column were carefully removed by pulling out the translucent plastic liner after laying the tube down on its side. The rooting profile was then divided into four equal sections (0–25, 25–50, 50–75, and 75–100 cm soil depths) by cutting from top to bottom with a large kitchen knife (Figure 1). Each root section was separately washed and placed into a labeled paper bag, so that root DM distribution could be determined by depth. All shoot and root samples were oven-dried in a forced air drier at 80°C until a constant weight was attained (typically 4 days) and then final shoot and root DM of each sample was recorded. Root-to-shoot DM ratio (R:S) was calculated as the ratio of root DM to shoot DM. Whole-plant water use efficiency (WUE; g L^–1^) was calculated as the ratio of whole-plant DM to total cumulative water use.

### Statistical Analyses

All statistical computations were performed using the PROC GLIMMIX procedure of SAS Version 9.4 (SAS Institute Inc., Cary, NC, United States). A Type 1 error rate of 0.05 was used for all statistical tests unless otherwise stated. Since the dependent variables whole-plant DM, shoot DM, root DM, R:S, water use, WUE, and VSWC were quantitative and continuous, a generalized linear mixed model was fitted with an identity link function and a Gaussian response distribution. The variances of whole-plant DM, shoot DM, root DM, R:S, water use, and WUE per plant were partitioned into the fixed effects of soil mix and watering treatments, and their interactions (soil mix × watering treatments), and the random effects of blocks. The following statistical model was used:

Y=ijkμ+w+is+jws+ijB+kεijk

where *Y*_*ijk*_ denotes the value of the measured trait for the *i*th watering treatment (mild water stress or control) of the *j*th soil mix treatment (0, 50, or 67% FS mixes) in the *k*th block, μ is the grand mean, *w*_*i*_ is the watering treatment effect (the first factor), *s*_*j*_ is the soil mix treatment effect (i.e., the second factor), *ws*_*ij*_ is the interaction effect between watering treatment and soil mix (watering treatment and soil mix, as well as their interaction, were considered as fixed effects), *B*_*k*_ is the effect of the *k*th block (treated as a random effect), and ε*_*ijk*_* is the residual.

The repeated measures analysis of variance of root DM and VSWC distribution by depth was partitioned into the fixed effects of soil mix treatments, watering treatments, and depth, and their interactions (soil mix × watering, soil mix × depth, watering × depth, and soil mix × watering × depth), and the random effects of blocks. Since the spacing interval between the root DM and VSWC distribution by depth measurements was equally spaced, the *Kenward-Roger* adjustment for bias correction for the denominator degrees of freedom was applied (Kenward and Roger, 1997). The fit of three possible types of repeated measure covariance structures [*compound symmetric*, CS; *autoregressive order 1*, AR(1); and heterogeneous autoregressive order 1, ARH(1)] were also compared and the most appropriate model was selected based on the AICC fit statistic, no overdispersion based on the generalized Chi-square/df, and assessment of conditional studentized residual plots. The random *subject* variance was modeled within the selected covariance structure. *F*-tests and log-likelihood ratio tests were used to determine the significance of fixed and random effects, respectively. Least square means were compared pairwise using Tukey’s test.

The confirmed assumptions of the analysis of variance were that the model effects were linear with an additive variance; the experimental errors were random, independent of treatment and design effects, and normally distributed about a zero mean with an equal variance. The homogeneity of error variance was tested by plotting the studentized residuals against factor levels and predicted values. The normality of error variance was tested by generating a Q-Q plot and scatterplots of studentized residuals against factor levels and predicted values, and by performing a formal test of normality using a Shapiro-Wilk. The linearity of fixed effects was evaluated by generating scatter and box plots of marginal and conditional residuals. Putative outliers, if any, were detected if the absolute values of the studentized residuals were not within the range of −3.4 to 3.4 (Bowley, 2015).

## Results

### Effects of Growth Medium and Water Stress on DM, Water Use, and WUE

Table 1 shows the effects of soil mix and watering treatments on final plant DM, water use, and WUE. Of the growth media tested, the highest whole-plant growth, water use, and WUE occurred with the 67% FS mix. Averaged across the two watering treatments, the 67% FS mix significantly increased (*p* < 0.0001) shoot DM by 123%, root DM by 81%, total DM by 112%, water use by 79%, and WUE by 21%, as compared to the 0% FS mix (Table 1). Although the water stress treatment was mild, it had a significant effect on every parameter measured. That is, averaged across the three growth media, the water stress treatment significantly reduced shoot DM by 24% (*p* < 0.001), root DM by 13% (*p* < 0.01), whole-plant DM by 22% (*p* < 0.001), and water use by 25% (*p* < 0.001). However, the water stress treatment significantly increased R:S by 18% (*p* < 0.01) and WUE (*p* < 0.05) by 6% (Table 1).

**TABLE 1:** A generalized linear mixed model analysis of the effects of soil mix, watering treatment, and soil mix by water interaction on final shoot dry matter (DM), root DM, whole-plant DM, root-to-shoot DM ratio (R:S), water use from planting to harvest, and whole-plant DM-based water use efficiency (WUE) of a single Ontario-adapted commercial soybean variety (*OAC Bayfield*) grown in a greenhouse under three growth media [67, 50, and 0% field soil (FS) mix] and two watering treatments [Control (100% soil water holding capacity; SWHC) and Stress (75% SWHC)] in 1-m rooting columns in 2015.

	Shoot DM (g plant^–1^)	Root DM (g plant^–1^)	Total DM (g plant^–1^)	Root-to-shoot DM ratio (g g^–1^)	Water use (L plant^–1^)	WUE (g L^–1^)
**Soil mix (S)**						
67% FS	7.3a^†^	2.2a	9.5a	0.31b	4.9a	1.98a
50% FS	5.0b	1.4b	6.4b	0.29b	3.9b	1.61b
0% FS	3.3c	1.2b	4.5c	0.40a	2.7c	1.63b
S.E.	0.28	0.07	0.34	0.012	0.14	0.051
*p* value	** < 0.0001**	** < 0.0001**	** < 0.0001**	** < 0.0001**	** < 0.0001**	** < 0.0001**
**Water (W)**						
Control	5.9a^†^	1.7a	7.6a	0.30b	4.4a	1.69b
Stress	4.5b	1.5b	6.0b	0.36a	3.3b	1.79a
S.E.	0.24	0.06	0.29	0.009	0.12	0.012
*p* value	**0.0001**	**0.0080**	**0.0002**	**0.0012**	**0.0001**	**0.0358**
S × W *p* value	0.2925	**0.0008**	0.0961	**0.0216**	0.0632	0.2777

*The plants were 46 days old at harvest. Six replicates were used. There was a two-way interaction (soil × water) effect for root DM and R:S. ^†^Within a factor (soil mix or water) and column, least-square means followed by the same letter are not significantly different (p ≥ 0.05) according to a Tukey’s test. Significant effects (p < 0.05) are indicated in bold.*

**FIGURE 3:**
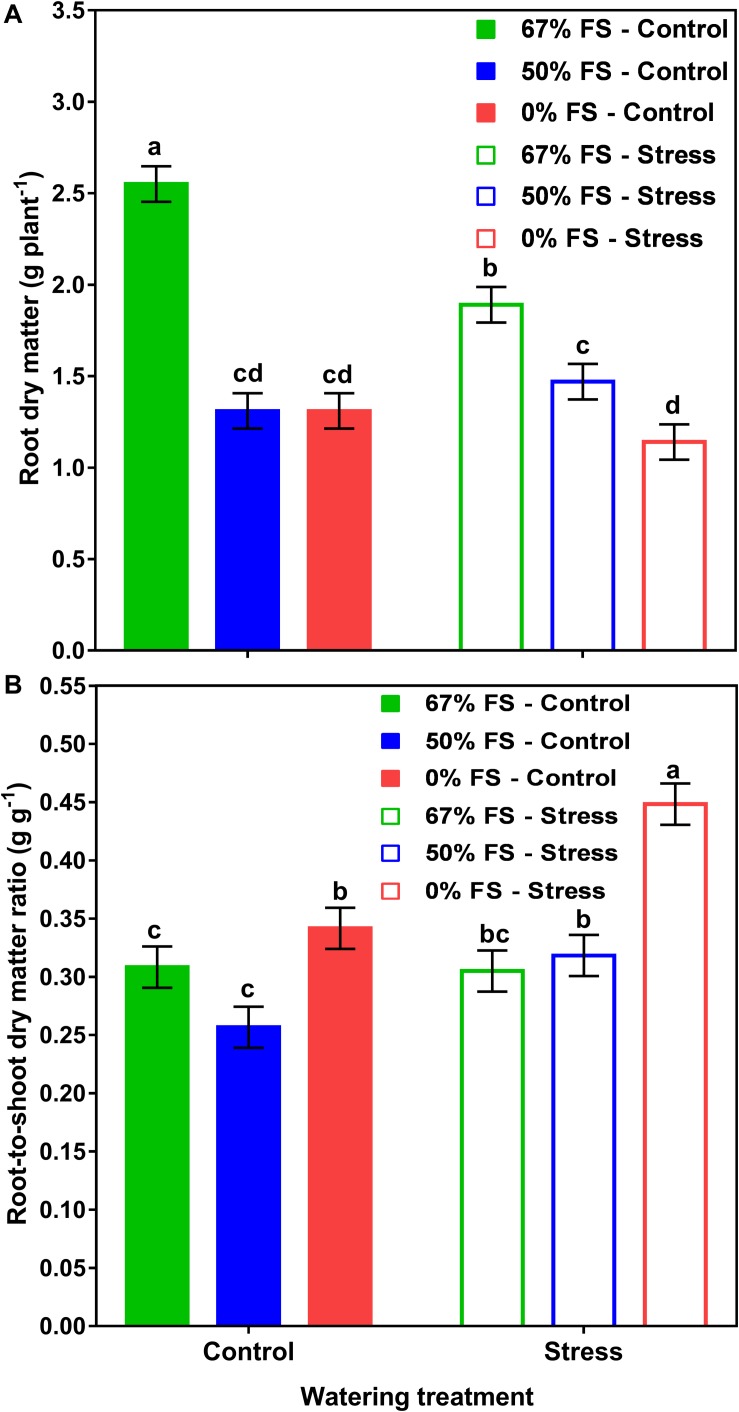
Interactive effects of growth medium and water stress treatments on root dry matter **(A)** and root-to-shoot dry matter ratio **(B)** for 46-day-old plants of the Ontario-adapted commercial soybean variety *OAC Bayfield*. Growth media treatments are 67% field soil (FS), 50% FS, or 0% FS mixes, watered daily to either 100% soil water holding capacity (SWHC; Control) or 75% SWHC (Stress) in 1-m rooting columns in a greenhouse environment. Data represent the soil mix by watering treatment interaction least square mean values ± 1 s.e.m. Six replicates were used. Within a measured trait (panel), soil mix by water stress treatment interaction least-square means followed by the same letter are not significantly different (*p* ≥ 0.05) according to a Tukey’s test.

Moreover, there was a significant soil mix by watering treatment interaction effect for root DM (*p* < 0.001) and R:S (*p* < 0.05) (Figure 3 and Table 1). When the growth media were 50 and 0% FS, the control and stress watering treatments did not differ for root DM but differed for R:S. However, when the growth medium was 67% FS, the root DM was significantly (*p* < 0.001) higher under the control watering treatment than under the stress treatment. There was no significant difference for R:S in the 67% FS growth medium between the control and stress watering treatments (Figure 3 and Table 1). Growth medium and watering treatments also affected final plant height and SPAD readings similarly to whole-plant DM (Supplementary Table 1).

**FIGURE 4:**
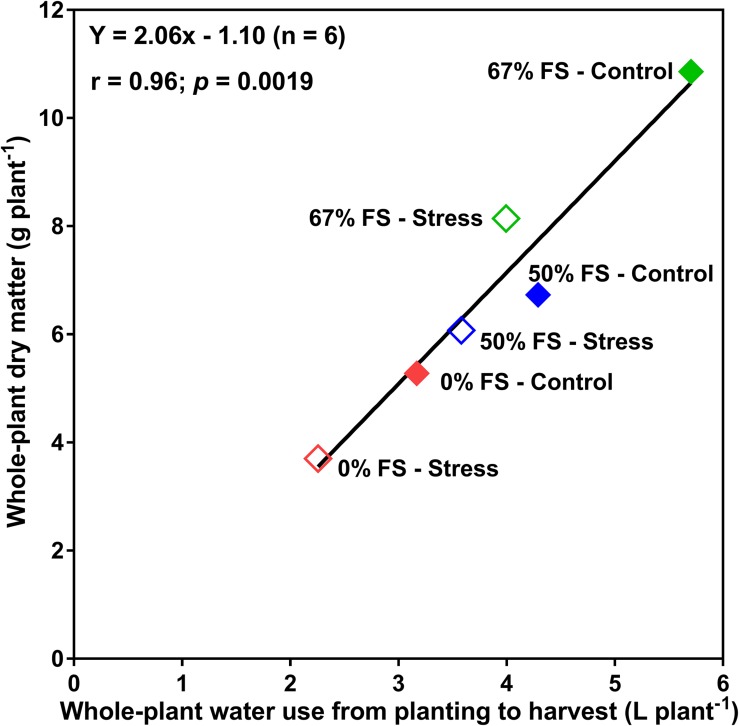
Relationship between whole-plant water use and the whole-plant dry matter for 46-day-old plants of the Ontario-adapted commercial soybean variety *OAC Bayfield*. Growth media treatments are 67, 50, or 0% FS mixes, watered daily to either 100% soil water holding capacity (SWHC; Control) or 75% SWHC (Stress). The line is the best-fit regression not forced through the origin. Data represent the soil mix by water stress treatment interaction least square mean values. Six replicates were used.

There was also a strong relationship (*r* = 0.96; *p* < 0.01) between whole-plant water use and final whole-plant DM accumulation (Figure 4). The 67% FS (the best performing growth medium) and the 0% FS mix (the poorest growth medium) had the highest and lowest, respectively, DM accumulation under both control and water stress conditions. The 50% FS mix had intermittent results under both control and water stress conditions.

### Effects of Growth Medium and Water Stress on VSWC Distribution by Depth

**FIGURE 5:**
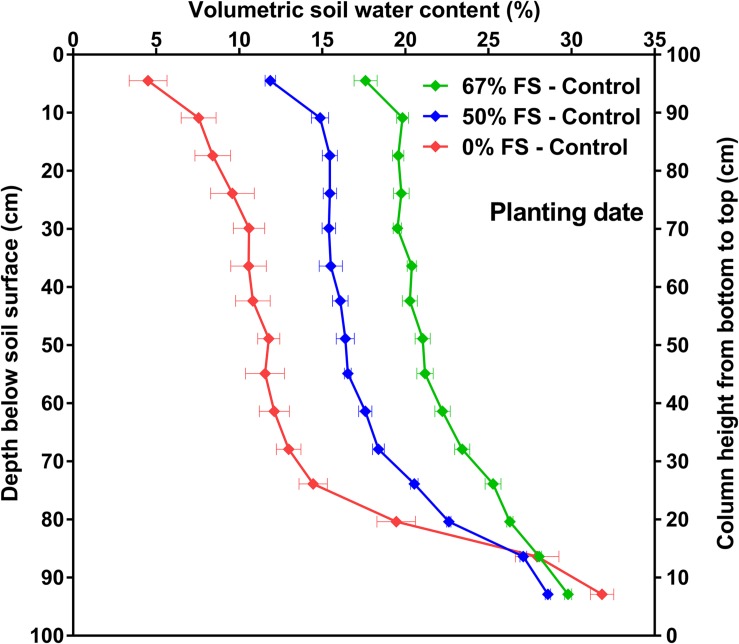
Volumetric soil water content (VSWC; %) by depth for the three growth media tested, measured at planting date. Growth media treatments are 67, 50 or 0% FS mixes, watered daily to 100% soil water holding capacity (Control). VSWC measurements were taken 24 h after the previous watering. Data are the means of 12 experimental units (tubes) ± 1 s.e.m. in each growth medium treatment. If not seen, the standard error is smaller than the symbol.

The VSWC in the rooting columns significantly (*p* < 0.0001) varied by depth in the soil profile throughout the experiment, very much as it does in a field situation (e.g., Figure 5 and Supplementary Figure 2). Initially, the variation in VSWC with depth was caused by the 0.01 MPa difference in gravitational potential between the bottom and top part of the rooting columns (Figure 5 and Supplementary Figure 2). Compared to the other growth media tested, the 67% FS mix under the control watering treatment resulted in a more uniform field-like VSWC over most of the 1 m depth similar to what is reported in the literature (Kirkham et al., 1998; Cutforth et al., 2013; Guo et al., 2016; Niu et al., 2017; Zhang et al., 2018), and thus the smallest change (∼12%) in VSWC between the bottom and top, perhaps with more available water for plant growth (Figure 5 and Supplementary Figure 2). For the same difference in gravitational potential, there was a larger change (∼27%) in VSWC and therefore probably less plant available water in the 0% FS mix, while the 50% FS mix (∼18%) fell in between the other two growth media (Figure 5 and Supplementary Figure 2).

**FIGURE 6:**
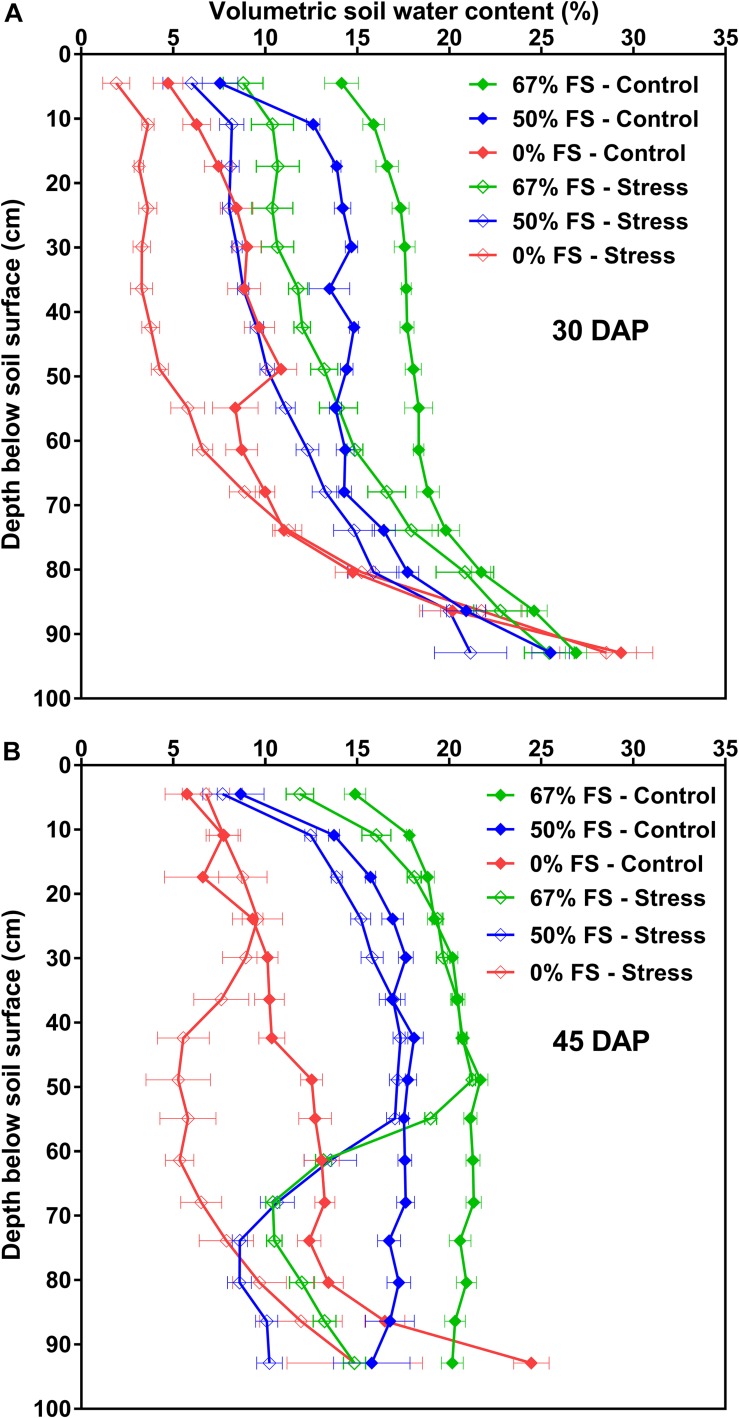
Volumetric soil water content (VSWC; %) by depth for the three growth media tested, measured at different days after planting (DAP): **(A)** (30 DAP; top), **(B)** (45 DAP; bottom). Growth media treatments are 67, 50 or 0% FS mixes, watered daily to either 100% soil water holding capacity (SWHC; Control) or 75% SWHC (Stress). VSWC measurements were taken 24 h after the previous watering. Data represent the soil mix by watering treatment interaction least square mean values ± 1 s.e.m. Six replicates were used. If not seen, the standard error is smaller than the symbol.

The changes in VSWC profile by depth over time for the three growth media under control and water stress watering treatments are also shown in Figure 6 and Supplementary Figure 3. Growth medium significantly (*p* < 0.0001) affected the VSWC profile by depth before and after water stress was imposed. There were clear differences in the VSWC profiles for the three growth media under control conditions. Moreover, the pattern of soil water depletion was consistent for the three growth media especially under control conditions; drier at the top, and the bottom also got drier over time. As expected, the water stress treatment (75% SWHC) resulted in a lower minimum VSWC, and this effect increased as plants grew larger and daily water use increased. Compared to all growth media tested, the 67% FS mix watered to 100% SWHC resulted in a more uniform soil water profile from top to bottom particularly when the plants were younger (at 30 and 35 DAP) but also over the entire experiment (Figure 6 and Supplementary Figure 3). Under stress conditions, the 67% FS mix had a more uniform VSWC profile with depth only at 30 DAP, when the plants were younger (Figure 6A). However, once the plants got older (35–45 DAP), the VSWC profile of the 67% FS mix under stress was not uniform from top to bottom; it was wetter at the upper top part but drier in the middle and bottom part of the profile (Figure 6B and Supplementary Figure 3).

**FIGURE 7:**
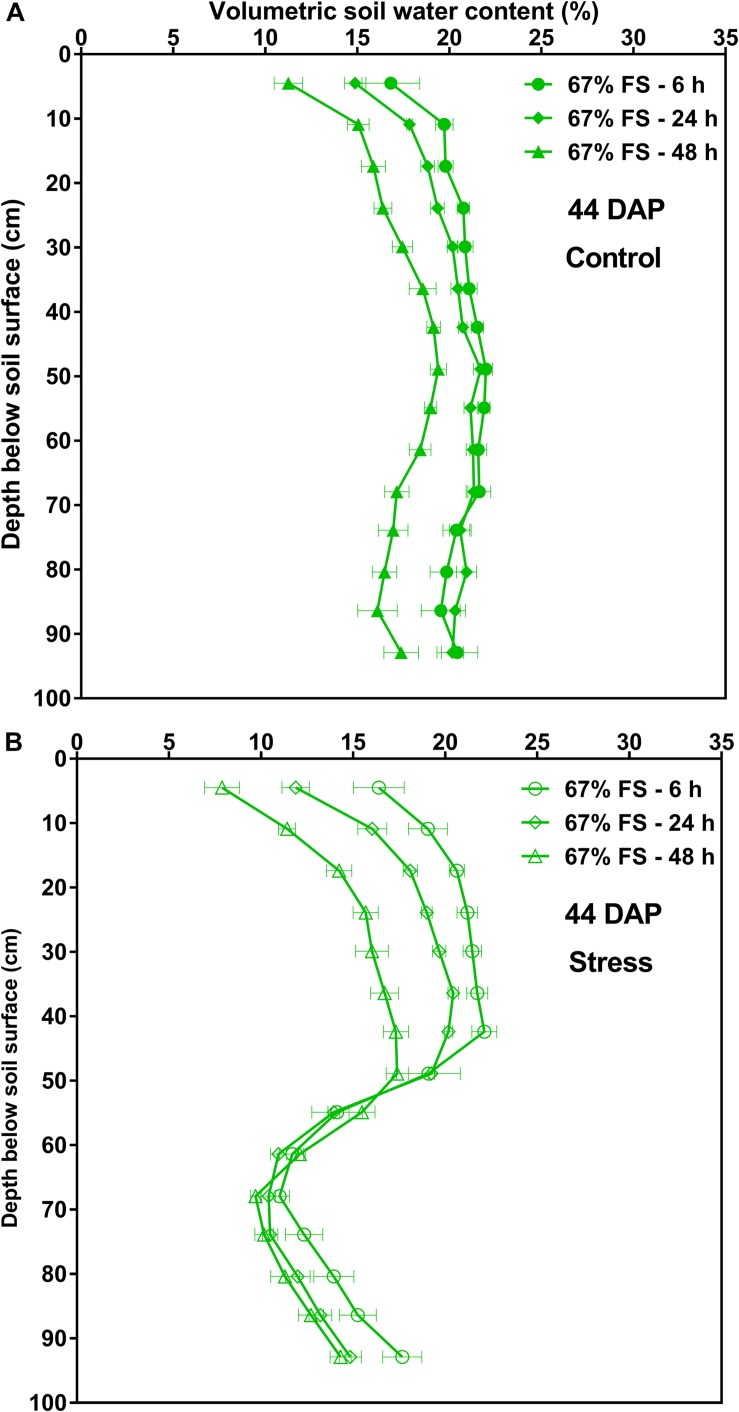
Volumetric soil water content by depth for the 67% field soil mix, measured at 6, 24, and 48 h after watering at 44 days after planting (44 DAP). **(A)** Represents tubes that were watered to 100% soil water holding capacity (SWHC; Control) whereas **(B)** represents the tubes that were watered to 75% SWHC (Stress). Data represent the least square mean values ± 1 s.e.m. in each water stress treatment over time. Six replicates were used. If not seen, the standard error is smaller than the symbol.

The changes in VSWC profile by depth over time for the 67% FS growth medium measured at 6, 24, and 48 h after watering are also shown in Figure 7 and Supplementary Figure 4 at two different DAP. Under control conditions (100% SWHC), there was uniform soil water depletion over time. The VSWC profile measured 24 h after watering was more uniform from top to bottom as compared to the 6 h and 48 h ones (Figure 7A and Supplementary Figure 4A). Under stress conditions (75% SWHC), the pattern of soil water depletion was different from that of the control. The soil water depletion was mostly from the top part of the profile as the bottom profile was drier and therefore the roots could not extract much water (Figure 7B and Supplementary Figure 4B).

### Effects of Growth Medium and Water Stress on Root DM Distribution by Depth

**FIGURE 8:**
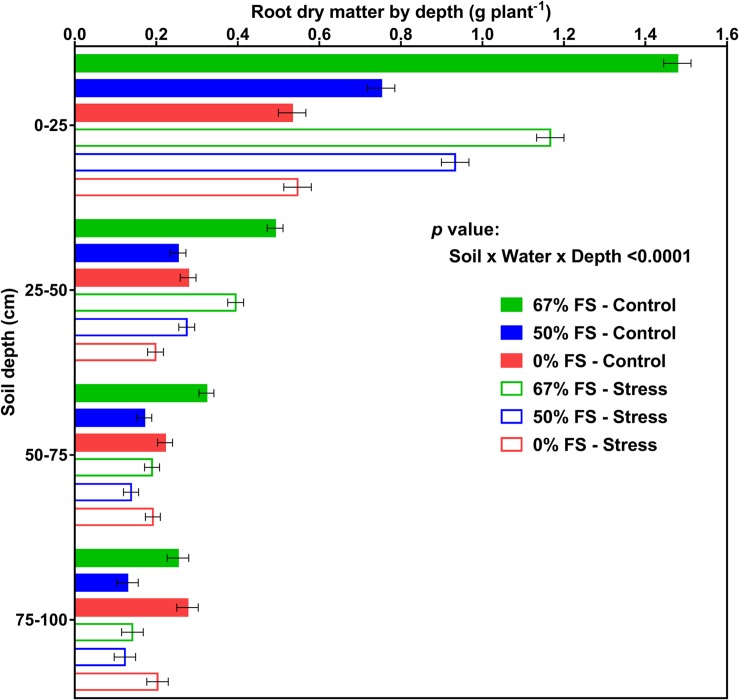
Effects of soil mix, watering treatment, and soil depth on root dry matter distribution for 46-day-old plants of the Ontario-adapted commercial soybean variety *OAC Bayfield*. Growth media treatments are 67, 50, or 0% FS mixes, watered daily to either 100% soil water holding capacity (SWHC; Control) or 75% SWHC (Stress). Data represent the soil mix × water × depth least square mean values ± 1 s.e.m. in each soil mix and water stress treatment. Six replicates were used. There was a three-way interaction (soil × water × depth) effect for root dry matter.

**FIGURE 9:**
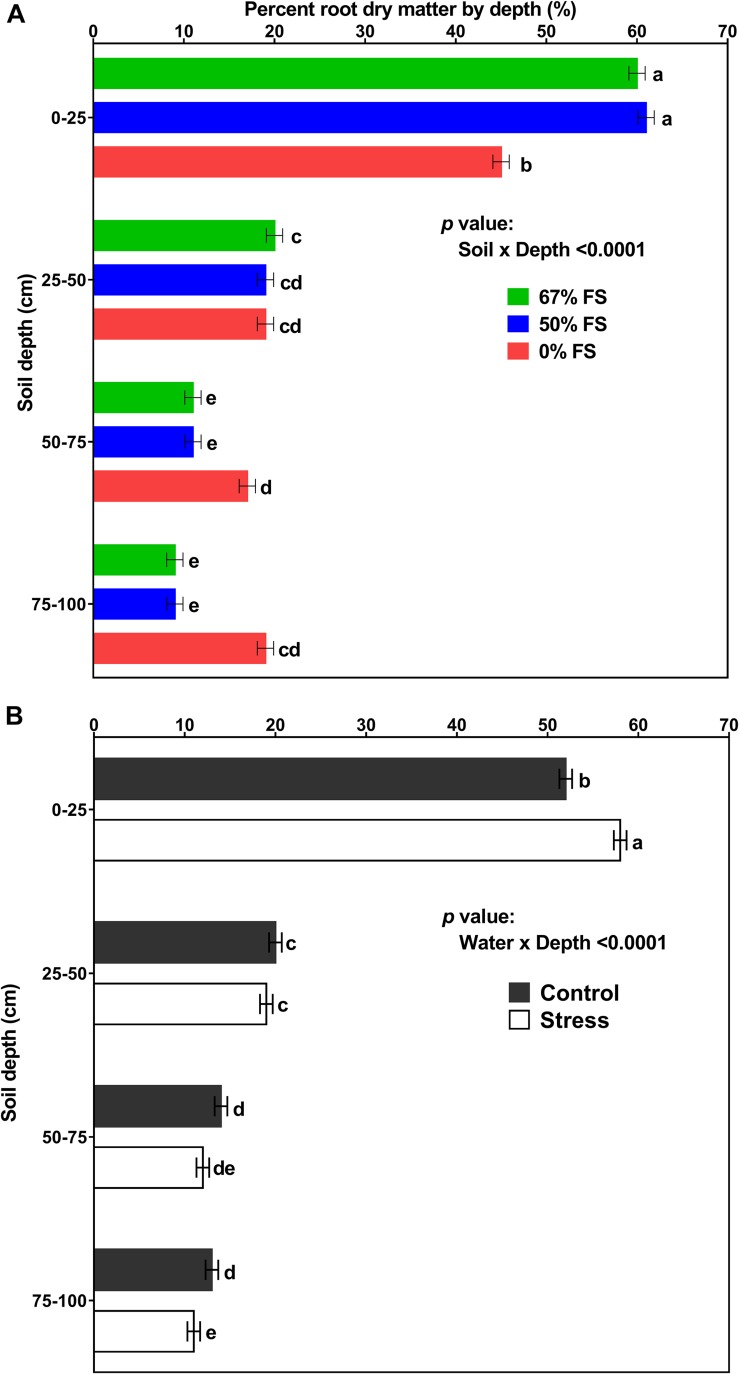
Effect of soil mix, watering treatment, and soil depth on percent root dry matter distribution for 46-day-old plants of the Ontario-adapted commercial soybean variety *OAC Bayfield*. Growth media treatments are 67, 50, or 0% FS mixes, watered daily to either 100% soil water holding capacity (SWHC; Control) or 75% SWHC (Stress). The top panel represents soil mix × depth (averaged across both watering treatments) and the bottom panel represents water × depth (averaged across three soil mixes) least square mean values ± 1 s.e.m. Six replicates were used. Within a soil column, growth media **(A)** or water stress **(B)** treatment least-square means labeled with the same letter are not significantly different (*p* ≥ 0.05) according to a Tukey’s test. If not seen, the standard error is smaller than the symbol. There was no significant three-way interaction (soil × water × depth) effect for percent root dry matter distribution.

Figures 8, 9 display the effects of soil mix, watering treatment, and soil depth on root and percent root DM distribution by depth. Of all the growth media tested, the 67% FS mix had the highest total root DM (Table 1) and exhibited a typical field-like rooting profile by depth similar to what is reported in the literature (Dwyer et al., 1988; Benjamin and Nielsen, 2006; Cutforth et al., 2013; Guo et al., 2016; Zhang et al., 2018). There were statistically significant effects of soil mix, watering treatment, depth, soil × water, soil × depth, and soil × water × depth for root DM (*p* < 0.0001) but there was no significant effect of water × depth (Supplementary Table 4). Furthermore, averaged across the three growth media and two watering treatments, there were statistically significant differences between the four profile depths. That is, a significantly higher amount of root DM was allocated to the top part of the profile (0–25 cm; 0.91 ± 0.014 g) followed by 25–50 cm (0.32 ± 0.009 g) and 50–75 cm (0.21 ± 0.009 g) depths, while the bottom part of the profile (75–100 cm depth) had the lowest amount of root DM (0.18 ± 0.011 g) (Supplementary Table 2).

The soil × water × depth interaction effect on root DM (*p* < 0.0001) is shown in Figure 8. When the growth medium was 0% FS mix, the control and stress watering treatments differed for root DM only at the 25–50 cm depth (greater root DM in the control). However, when the growth medium was 67% FS mix, root DM was significantly (*p* < 0.0001) higher under the control than the stress treatment at all depths of the soil profile (0–25, 25–50, 50–75, and 75–100 cm) (Figure 8).

Furthermore, there were statistically significant effects of depth, soil × depth, and water × depth for percent root DM distribution (*p* < 0.0001). However, there were no significant effects of soil mix, watering treatment, soil × water, and soil × water × depth for percent root DM distribution (Supplementary Table 5).

Averaged across the three growth media and two watering treatments, a significantly (*p* < 0.0001) higher percentage root DM was allocated to the top part of the profile (0–25 cm; ∼55%) followed by the 25–50 cm (∼20%) profile depths. The two bottom profile depths, 50–75 cm, and 75–100 cm did not differ from each other, each contributing about 13% of the root DM (Supplementary Table 3).

Averaged across the two watering treatments, the 67% FS mix had a significantly (*p* < 0.0001) higher percent root DM in the top part of the profile (0–25 cm profile depth; ∼60%) as compared to the 0% FS mix (∼45%). The 0% FS mix, however, had a significantly greater percentages of root DM distributed in the bottom parts of the soil profile [(50–75 cm; ∼17%) and (75–100 cm; ∼19%)], while there was no difference between the three growth media at 25–50 cm depth of the soil profile (Figure 9A).

Averaged across the three growth media, the water stress treatment had a significantly (*p* < 0.0001) greater proportion (∼58%) of root DM in the top part of the soil profile (0–25 cm) as compared to the control watering treatment (∼52%), while there was no difference between the two watering treatments at 25–50 and 50–75 cm depths of the soil profile. The control watering treatment, however, had a significantly greater proportion (∼13%) of root DM in the bottom part of the soil profile (75–100 cm) as compared to the stress watering treatment (∼11%) (Figure 9B).

## Discussion

The present study produced a unique culture system for controlled environment phenotyping. It employs 1 m tall, 10 cm diameter rooting columns and a 67% FS growing medium that provides for relatively uniform VSWC throughout the profile to support plant growth, with realistic field-like soil water and rooting profile variation with depth.

We predicted that the soil mix with the highest fraction of FS (the 67% FS mix) in 1-m rooting columns would be the best growth medium resulting in higher DM, water use, WUE, and uniform (field-like) VSWC and rooting profile variation with depth than the soil mixes containing less FS, under control and stress conditions. The highest soybean growth (whole-plant DM and water use) was observed in the 67% FS mix followed by the 50% FS mix whereas the least performing growth medium for those parameters was the 0% FS mix. The better performance of the plants under the former two growth media could be because these growth media with higher ratios of field soil (i.e., the 50 and 67% FS amended mixes) may have greater SWHC since water is held more tightly in the micro pores of the field soil component. However, the best performing growth media of all the three soil mixes examined was the 67% FS mix. This finding is consistent with our prediction supporting our hypothesis. Additionally, the plants in the 67% FS mix also had higher WUE (21% higher than that of the 0% FS mix). This higher WUE could be attributed to higher SPAD readings displayed in the 67% FS mix (Supplementary Table 1) as compared to the other two soil mixes. This suggests the 67% FS growth medium may have had improved nutrient status, resulting in taller, healthier, and greener plants that had a stronger photosynthetic response to leaf internal CO_2_ concentration, although we did not perform photosynthetic gas exchange measurements on these plants. Unfortunately, in comparing the effects of growth medium on plant DM accumulation in the present study, it is not possible to separate the effects of nutrient status from the direct effects of SWHC. However, given the large effects of growth medium on water use (Table 1) and the relatively small effects on SPAD values (Supplementary Table 1), it seems likely that the effects of SWHC predominate.

We also predicted that watering the rooting columns to 75% rather than 100% SWHC would benefit plant growth in the heavier textured (67% FS) soil mix (as there would not be enough air space in the soil watered to 100% SWHC), but would be detrimental to plant growth in the soil mix with no FS added to it (0% FS). However, our results do not support this prediction, as the 75% SWHC watering treatment did not benefit plant growth in any soil mix regardless of its composition. For every parameter we measured, the plants were more productive when they were fully watered to 100% SWHC (control) than to 75% SWHC (stress). For instance, under stress, the 67 and 0% FS mixes reduced whole-plant DM accumulation by 25 and 30%, respectively, indicating that in both cases the 75% SWHC watering treatment was a physiologically relevant stress level. We also predicted that mild water stress would increase R:S and WUE of soybean in this culture system. Indeed, the mild water stress treatment increased R:S and WUE by 18 and 6% (Table 1), indicating a greater proportion of DM allocation to the roots and more efficient utilization of available water under water stress conditions. Similar effects of water stress on R:S were obtained by Boutraa et al. (2010); Ehdaie et al. (2012), and Wang et al. (2014).

Whole-plant water use strongly correlated (*r* = 0.96) with whole-plant DM accumulation across all growth media and watering treatments (Figure 4), suggesting that water use measurement can serve as a reasonable proxy for real-time DM accumulation (growth) without requiring a destructive harvest. This very strong association between water use and biomass production also suggests the effects of growth medium treatments on whole-plant DM were consistent with effects on water use.

We also explored which growth medium in deep-rooting columns under controlled environment conditions could provide more realistic VSWC and rooting profile variation with depth, similar to field soil conditions. We predicted that growth media with higher ratios of FS (i.e., the 50 and 67% FS mixes) would have a more uniform VSWC profile with depth since sandy soils (0% FS mix) generally have a larger change in VSWC between 0.00 and 0.01 MPa water potential. As predicted, the 67% FS mix displayed a realistic and quite uniform VSWC profile for most of its depth (e.g., Figures 5, 6 and Supplementary Figures 2, 3) emulating the typical field VSWC profiles reported in the literature. Compared to our findings, similar field VSWC profiles by depth have been obtained in soybean (Dwyer et al., 1988; Kirkham et al., 1998; Benjamin and Nielsen, 2006), maize [*Zea mays* L. (Dwyer et al., 1988; Kirkham et al., 1998; Zhang et al., 2018)], chickpea [*Cicer arietinum* L. (Cutforth et al., 2013; Gan et al., 2015; Niu et al., 2017)], dry bean [*Phaseolus vulgaris* L. (Nuñez Barrios et al., 2005)], field pea [*Pisum sativum* L. (Cutforth et al., 2013; Gan et al., 2015; Niu et al., 2017)], canola [*Brassica napus* L. (Cutforth et al., 2013)], sunflower [*Helianthus annuus* L. (Meinke et al., 1993)], wheat [*Triticum aestivum* L. (Cutforth et al., 2013; Gan et al., 2015; Guo et al., 2016; Niu et al., 2017)], lentil [*Lens culinaris* Medik. (Gan et al., 2015; Niu et al., 2017)], and barley [*Hordeum vulgare* L. (Dwyer et al., 1988; Gan et al., 2015)].

The uniformity of the VSWC profile with depth for the three growth media clearly differed under control and water stress conditions although the pattern of soil water depletion was consistent for the three growth media especially under control conditions: relatively higher VSWC at the upper and bottom parts of the soil profile over time in the growth media with higher fraction of field soil. Conversely, the 0% FS mix had a drier profile at the top and wetter profile at the bottom, regardless of the watering treatment and the stage of the crop. Specifically, for the 67% FS mix, the VSWC profile was quite uniform over most of its depth under control conditions, especially later in the experiment (e.g., at 35–45 DAP). This might be due to (1) later in the developmental stage of the crop, the root systems reached down to the bottom part of the profile and became more effective at extracting the available soil water at depth, (2) the profile did not really get back to equilibrium against gravity, because as water was being added to the top, it never completely wetted the bottom since it got intercepted and taken up by the plant roots before it got there. Thus, the bottom part of the profile got a little bit drier over time but in general, was similar to what is observed in the field. Still, across all growth media tested, most water depletion seemed to have occurred from the upper part of the profile where most of the roots were distributed. The water stress treatment, however, created a much less uniform VSWC profile with depth. It produced a profile with a smaller change in VSWC at the top but a bigger change in VSWC at the bottom. This could be because the water added at the top kept the upper profile wetter but the water never made it to the bottom part of the profile. This stress experienced mostly by the lower roots was strong enough to reduce whole-plant DM by 25%.

Figure 7 and Supplementary Figure 4 also show how the VSWC profile evolved over the 48-hour period after watering for the 67% FS mix. The soil water depletion was quite uniformly distributed across the height of the tubes under control conditions (Figure 7A and Supplementary Figure 4A). This could be due to the presence of roots and plant-available water well distributed from top to the bottom part of the profile. Contrarily, most of the soil water depletion occurred at the top, wetter part of the soil profile under stress conditions (Figure 7B and Supplementary Figure 4B), even though roots were present in the deeper sections. This likely occurred because the deeper roots had already reduced the soil water to near the wilting point in the lower profile, and since this layer was never re-wet by water additions at the top of the tube, soil water extraction ceased.

In agreement with our prediction, the rooting profiles observed in this study were quite similar to those that have been observed in the field, where the majority of the roots (>50%) are located in the upper (e.g., 0–25 cm) soil layer under both control and water stress conditions [soybean (Dwyer et al., 1988; Kirkham et al., 1998; Benjamin and Nielsen, 2006; Ordonez et al., 2018)], maize (Dwyer et al., 1988; Kirkham et al., 1998; Ordonez et al., 2018; Zhang et al., 2018), field pea (Cutforth et al., 2013), canola (Cutforth et al., 2013), wheat (White and Kirkegaard, 2010; Cutforth et al., 2013; Kang et al., 2014; Wang et al., 2014; Guo et al., 2016; Hodgkinson et al., 2017), and barley (Dwyer et al., 1988)]. Growth medium also strongly affected root DM distribution by depth, with a higher fraction of root DM distributed in the deeper soil layers in the 0% FS growth medium treatment.

In this study, although the growth medium treatment with the highest fraction of field soil (67% FS mix) was the best growth medium, it proved impractical to increase the percentage of field soil much above this level. For example, in preliminary experiments we found that using 100% FS mix as the growth medium resulted in excessive slumping (up to 25 cm) of the soil column over the course of the experiment, even though we took care to load and pack the columns in stages. Tubes with 100% FS mix (19% clay texture) were also susceptible to surface crusting due to slaking under daily wetting and drying. It is possible that a soil with lower clay content would not have exhibited this defect (Valentin and Ruiz Figueroa, 1987).

## Conclusion

In conclusion, the 0% FS growth medium, previously used successfully in small pot culture of soybean, proved unsuitable in 1-m rooting columns because VSWC dropped too drastically with height above free drainage. In the same 1-m rooting columns, a growth medium that included 67% FS mix provided reasonable field-like soil water and rooting profiles by depth. This culture system could be useful to simulate field soil water and rooting profiles, water stress, and growth responses of soybean and other crops under controlled environment conditions. Increasing the fraction of field soil increased SWHC, and increased the uniformity of VSWC with depth. Our results confirmed that the plants responded to water stress differently in different growth media. In addition, although the 75% SWHC watering treatment was a mild water stress, it was a physiologically relevant stress level that reduced shoot, root, and whole-plant DM accumulation each by about 25%. In general, plant DM accumulation, water use, and WUE were the highest in the growth medium that contained the highest fraction of field soil. Therefore, these overall results suggest the 67% FS mix as the best growth medium for controlled environment studies using these rooting columns. This study provides novel phenotyping tools to select for root function and yield formation traits that could decrease soybean yield losses under soil water deficit conditions. It should also be noted that this culture system was developed for soybean growth but it may also be appropriate for other important agronomic field crops in the region such as corn, wheat, dry bean, canola, and barley.

## Data Availability Statement

The datasets generated and analyzed for this study are available on request to the corresponding author.

## Author Contributions

MG performed the experiment, data collection, statistical data analysis and presentation, and drafted the manuscript. Both authors conceived the project and experimental design and collaborated on the data interpretation and manuscript revision.

## Conflict of Interest

The authors declare that the research was conducted in the absence of any commercial or financial relationships that could be construed as a potential conflict of interest.

## References

[B1] Benjamin J. G. Nielsen D. C. (2006). Water deficit effects on root distribution of soybean, field pea and chickpea. *Field Crops Res.* 97 248–253. 10.1016/j.fcr.2005.10.005

[B2] Boutraa T. Akhkha A. Al-Shoaibi A. A. Alhejeli A. M. (2010). Effect of water stress on growth and water use efficiency (WUE) of some wheat cultivars (Triticum durum) grown in Saudi Arabia. *J. Taibah Univer. Sci.* 3 39–48. 10.1016/s1658-3655(12)60019-3

[B3] Bowley S. R. (2015). *A Hitchhiker’s Guide To Statistics In Biology: Generalized Linear Mixed Model Edition.* Kincardine: University of Guelph.

[B4] Chard J. K. Bugbee B. (2005). *Simulating the Field: How to Grow Plants in Soil Columns in the Greenhouse.* Logan, UT: Utah State University.

[B5] Cutforth H. W. Angadi S. V. McConkey B. G. Miller P. R. Ulrich D. Gulden R. (2013). Comparing rooting characteristics and soil water withdrawal patterns of wheat with alternative oilseed and pulse crops grown in the semiarid Canadian prairie. *Can. J. Soil Sci.* 93 147–160. 10.4141/cjss2012-081

[B6] Dwyer L. M. Stewart D. W. Balchin D. (1988). Rooting Characteristics of Corn, Soybeans and Barley as a Function of Available Water and Soil Physical Characteristics. *Can. J. Soil Sci.* 68 121–132. 10.4141/cjss88-011

[B7] Earl H. J. (2002). Stomatal and non-stomatal restrictions to carbon assimilation in soybean (Glycine max) lines differing in water use efficiency. *Environ. Exp. Bot.* 48 237–246. 10.1016/s0098-8472(02)00041-2

[B8] Earl H. J. (2003). A precise gravimetric method for simulating drought stress in pot experiments. *Crop Sci.* 43 1868–1873. 10.2135/cropsci2003.1868

[B9] Earl H. J. (2012). Drought stress in Ontario soybean – what’s really going on? *Paper Presented at the Physiology-Based Strategies For Sustainable Yield And Quality, ASA, CSSA, and SSSA Annual Meetings*, Cincinnati, OH.

[B10] Ehdaie B. Layne A. P. Waines J. G. (2012). Root system plasticity to drought influences grain yield in bread wheat. *Euphytica* 186 219–232. 10.1007/s10681-011-0585-9

[B11] Fredlund M. D. Wilson G. W. Fredlund D. G. (2002). Use of the grain-size distribution for estimation of the soil-water characteristic curve. *Can. Geotechn. J.* 39 1103–1117. 10.1139/t02-049

[B12] Gan Y. Hamel C. O’Donovan J. T. Cutforth H. Zentner R. P. Campbell C. A. (2015). Diversifying crop rotations with pulses enhances system productivity. *Sci. Rep.* 5:14625. 10.1038/srep14625 PMC4589733 26424172

[B13] Guo F. Ma J.-J. Zheng L.-J. Sun X.-H. Guo X.-H. Zhang X. (2016). Estimating distribution of water uptake with depth of winter wheat by hydrogen and oxygen stable isotopes under different irrigation depths. *J. Integr. Agricul.* 15 891–906. 10.1016/s2095-3119(15)61258-8

[B14] Hodgkinson L. Dodd I. C. Binley A. Ashton R. W. White R. P. Watts C. W. (2017). Root growth in field-grown winter wheat: Some effects of soil conditions, season and genotype. *Eur. J. Agron.* 91 74–83. 10.1016/j.eja.2017.09.014 29129966 PMC5669304

[B15] Hufstetler E. V. Boerma H. R. Carter T. E. Earl H. J. (2007). Genotypic variation for three physiological traits affecting drought tolerance in soybean. *Crop Sci.* 47 25–35. 10.2135/cropsci2006.04.0243

[B16] Jones H. G. (2007). Monitoring plant and soil water status: established and novel methods revisited and their relevance to studies of drought tolerance. *J. Exp. Bot.* 58 119–130. 10.1093/jxb/erl118 16980592

[B17] Kang L. Y. Yue S. C. Li S. Q. (2014). Effects of Phosphorus Application in Different Soil Layers on Root Growth, Yield, and Water-Use Efficiency of Winter Wheat Grown Under Semi-Arid Conditions. *J. Integrat. Agricult.* 13 2028–2039. 10.1016/s2095-3119(14)60751-6

[B18] Kenward M. G. Roger J. H. (1997). Small Sample Inference for Fixed Effects from Restricted Maximum Likelihood. *Biometrics* 53 983–997. 9333350

[B19] Kirkham M. B. Grecu S. J. Kanemasu E. T. (1998). Comparison of minirhizotrons and the soil-water-depletion method to determine maize and soybean root length and depth. *Eur. J. Agron.* 8 117–125. 10.1016/s1161-0301(97)00019-1

[B20] Meinke H. Hammer G. L. Want P. (1993). Potential soil-water extraction by sunflower on a range of soils. *Field Crops Res.* 32 59–81. 10.1016/0378-4290(93)90021-e

[B21] Niu Y. Bainard L. D. Bandara M. Hamel C. Gan Y. (2017). Soil residual water and nutrients explain about 30% of the rotational effect in 4-yr pulse-intensified rotation systems. *Can. J. Plant Sci.* 97 852–864.

[B22] Nuñez Barrios A. Hoogenboom G. Nesmith D. S. (2005). Drought sress and the distribution of vegetative and reproductive traits of a bean cultivar. *Sci. Agric.* 62 18–22. 10.1590/s0103-90162005000100004

[B23] OMAFRA, (2019). *Ontario Ministry of Agriculture, Food and Rural Affairs (OMAFRA). Estimated Area, Yield, Production And Farm Value Of Specified Field Crops, Ontario, 2012-2019, (Imperial and Metric Units).* Available at: http://www.omafra.gov.on.ca/english/stats/crops/index.html (accessed September 12, 2019).

[B24] Ordonez R. A. Castellano M. J. Hatfield J. L. Helmers M. J. Licht M. A. Liebman M. (2018). Maize and soybean root front velocity and maximum depth in Iowa, USA. *Field Crops Res.* 215 122–131. 10.1016/j.fcr.2017.09.003

[B25] Pang J. Turner N. C. Du Y.-L. Colmer T. D. Siddique K. H. M. (2017). Pattern of water use and seed yield under terminal drought in chickpea genotypes. *Front. Plant Sci.* 8:1375. 10.3389/fpls.2017.01375 28848579 PMC5552816

[B26] Passioura J. B. (2006). The perils of pot experiments. *Funct. Plant Biol.* 33 1075–1079. 10.1071/FP06223 32689318

[B27] Ray J. D. Sinclair T. R. (1997). Stomatal closure of maize hybrids in response to drying soil. *Crop Sci.* 37 803–807. 10.2135/cropsci1997.0011183x003700030018x

[B28] Ray J. D. Sinclair T. R. (1998). The effect of pot size on growth and transpiration of maize and soybean during water deficit stress. *J. Exp. Bot.* 49 1381–1386. 10.1093/jxb/49.325.1381

[B29] Sinclair T. R. Manandhar A. Shekoofa A. Rosas-Anderson P. Bagherzadi L. Schoppach R. (2017). Pot binding as a variable confounding plant phenotype: theoretical derivation and experimental observations. *Planta* 245 729–735. 10.1007/s00425-016-2641-0 27999989

[B30] Townend J. Dickinson A. L. (1995). A comparison of rooting environments in containers of different sizes. *Plant Soil* 175 139–146. 10.1007/bf02413019

[B31] Tuller M. Or D. Hillel D. (2004). Retention of water in soil and the soil water characteristic curve. *Encyclop. Soils Environ.* 4 278–289.

[B32] Valentin C. Ruiz Figueroa J. F. (1987). “Effects of kinetic energy and water application rate on the development of crusts in a fine sandy loam soil using sprinkling irrigation and rainfall simulation,” in *Micromorphologie des Sols, Soil Micromorphology*, eds Fedoroff N. Bresson L. M. Courty M. A. (Paris: AFES), 401–408.

[B33] van Genuchten M. T. (1980). A closed-form equation for predicting the hydraulic conductivity of unsaturated soils. *Soil Sci. Soc. Am. J.* 44 892–898. 10.2136/sssaj1980.03615995004400050002x

[B34] Wang C. Liu W. Li Q. Ma D. Lu H. Feng W. (2014). Effects of different irrigation and nitrogen regimes on root growth and its correlation with above-ground plant parts in high-yielding wheat under field conditions. *Field Crops Res.* 165 138–149. 10.1016/j.fcr.2014.04.011

[B35] White R. G. Kirkegaard J. A. (2010). The distribution and abundance of wheat roots in a dense, structured subsoil - implications for water uptake. *Plant Cell Environ.* 33 133–148. 10.1111/j.1365-3040.2009.02059.x 19895403

[B36] Zhang Y. L. Li T. T. Bei S. K. Zhang J. L. Li X. L. (2018). Growth and distribution of maize roots in response to nitrogen accumulation in soil profiles after long-term fertilization management on a calcareous Soil. *Sustainability* 10 1–16. 10.3390/su1011431530607262

