# Supplementary material for: Effects of Growth Medium and Water Stress on Soybean [Glycine max (L.) Merr.] Growth, Soil Water Extraction and Rooting Profiles by Depth in 1-m Rooting Columns
Source: Front Plant Sci. 2020 Apr 24;11:487. doi: 10.3389/fpls.2020.00487 (PMC7250135; doi:10.3389/fpls.2020.00487)
Supplement: Supplementary file 1 [file Data_Sheet_1.docx]

Supplementary Material

Supplementary Table 1. A generalized linear mixed model analysis of the effects of soil mix, watering treatment, and soil mix by water interaction on SPAD readings and plant height. Both parameters were measured at the harvest date (46 days after planting) for the Ontario-adapted commercial soybean variety *OAC Bayfield* grown in a greenhouse under three growth media [67, 50, and 0% field soil (FS) mix] and two watering treatments [Control (100% soil water holding capacity; SWHC) and Stress (75% SWHC) conditions] in 1-m rooting columns. Six replicates were used.

|  | SPAD | Height (cm) |
| --- | --- | --- |
| Soil mix (S) |  |  |
| 67% FS | 38.9 a**^†^** | 107.9 a |
| 50% FS | 37.1 b | 94.2 b |
| 0% FS | 34.1 c | 89.8 c |
| S.E. | 0.62 | 1.14 |
| *p* value | **<0.0001** | **<0.0001** |
|  |  |  |
| Water (W) |  |  |
| Control | 37.6 a**^†^** | 103.3 a |
| Stress | 35.8 b | 91.3 b |
| S.E. | 0.53 | 0.87 |
| *p* value | **0.0098** | **<0.0001** |
|  |  |  |
| S**×**W *p* value | 0.0870 | 0.1043 |

**^†^**Within a factor (soil mix or water) and column, least-square means followed by the same letter are not significantly different (*p* ≥ 0.05) according to a Tukey’s test. Significant effects (*p* < 0.05) are indicated in **bold.**

Supplementary Table 2. A generalized linear mixed model repeated measures analysis of the effects of soil depth on root dry matter (DM) for the Ontario-adapted commercial soybean variety *OAC Bayfield* grown in a greenhouse under three growth media [67, 50, and 0% field soil (FS) mix] and two watering treatments [Control (100% soil water holding capacity; SWHC) and Stress (75% SWHC) conditions] in 1-m rooting columns. Data represent the depth (averaged across three growth media and two watering treatments) least-square means ± 1 s.e.m. at each soil depth. Six replicates were used.

| Soil depth (cm) | Root DM (g plant^-1^) | Standard error |
| --- | --- | --- |
| 0-25 | 0.91 a**^†^** | 0.014 |
| 25-50 | 0.32 b | 0.001 |
| 50-75 | 0.21 c | 0.001 |
| 75-100 | 0.18 d | 0.011 |

**^†^**Within a column, least-square means followed by the same letter are not significantly different (*p* ≥ 0.05) according to a Tukey’s test.

Supplementary Table 3. A generalized linear mixed model repeated measures analysis of the effects of soil depth on percent root dry matter (DM) distribution for the Ontario-adapted commercial soybean variety *OAC Bayfield* grown in a greenhouse under three growth media [67, 50, and 0% field soil (FS) mix] and two watering treatments [Control (100% soil water holding capacity; SWHC) and Stress (75% SWHC) conditions] in 1-m rooting columns. Data represent the depth (averaged across three growth media and two watering treatments) least square mean values ± 1 s.e.m. at each soil depth. Six replicates were used.

| Soil depth (cm) | Root DM (%) | Standard error |
| --- | --- | --- |
| 0-25 | 55.3 a**^†^** | 0.52 |
| 25-50 | 19.5 b | 0.52 |
| 50-75 | 13.1 c | 0.52 |
| 75-100 | 12.1 c | 0.52 |

**^†^**Within a column, least-square means followed by the same letter are not significantly different (*p* ≥ 0.05) according to a Tukey’s test.

Supplementary Table 4. A generalized linear mixed model repeated measures analysis of the effects of soil mix, watering treatment, and soil depth on root dry matter for the Ontario-adapted commercial soybean variety *OAC Bayfield* grown in a greenhouse under three growth media [67, 50, and 0% field soil (FS) mix] and two watering treatments [Control (100% soil water holding capacity; SWHC) and Stress (75% SWHC) conditions] in 1-m rooting columns.

| Random effects | Subject | Estimate | Standard error | ChiSq | Pr > ChiSq**^†^** |
| --- | --- | --- | --- | --- | --- |
| Block (B) |  | 0.00011 | 0.000290 | 0.19 | 0.6656 |
| Var(1) | B**×**S**×**W | 0.00405 | 0.001155 |  |  |
| Var(2) | B**×**S**×**W | 0.00190 | 0.000580 |  |  |
| Var(3) | B**×**S**×**W | 0.00215 | 0.000543 |  |  |
| Var(4) | B**×**S**×**W | 0.00668 | 0.001743 |  |  |
| ARH(1) | B**×**S**×**W | 0.54470 | 0.100400 |  |  |
|  |  |  |  |  |  |
| Fixed effects | Num df | Den df | F value | Pr > F**^†^** |  |
| Soil mix (S) | 2 | 22.12 | 117.68 | **<0.0001** |  |
| Water (W) | 1 | 22.12 | 14.96 | **0.0008** |  |
| S**×**W | 2 | 22.12 | 17.45 | **<0.0001** |  |
| Depth (D) | 3 | 54.59 | 929.38 | **<0.0001** |  |
| S**×**D | 6 | 62.78 | 84.67 | **<0.0001** |  |
| W**×**D | 3 | 54.59 | 0.39 | 0.7609 |  |
| S**×**W**×**D | 6 | 62.78 | 10.25 | **<0.0001** |  |

**^†^**Significant effects (*p* < 0.05) are indicated in **bold**.

Supplementary Table 5. A generalized linear mixed model repeated measures analysis of the effects of soil mix, watering treatment, and soil depth on percent root dry matter distribution for the Ontario-adapted commercial soybean variety *OAC Bayfield* grown in a greenhouse under three growth media [67, 50, and 0% field soil (FS) mix] and two watering treatments [Control (100% soil water holding capacity; SWHC) and Stress (75% SWHC) conditions] in 1-m rooting columns.

| Random effects | Subject | Estimate | Standard error | ChiSq | Pr > ChiSq**^†^** |
| --- | --- | --- | --- | --- | --- |
| Block (B) |  | 0.00000 | **.** | 0.000 | 1.0000 |
| AR(1) | B**×**S**×**W | -0.15830 | 0.126400 |  |  |
| Residual |  | 0.00096 | 0.000128 |  |  |
|  |  |  |  |  |  |
| Fixed effects | Num df | Den df | F value | Pr > F**^†^** |  |
| Soil mix (S) | 2 | 45.35 | 0.00 | 1.0000 |  |
| Water (W) | 1 | 45.35 | 0.00 | 1.0000 |  |
| S**×**W | 2 | 45.35 | 0.00 | 1.0000 |  |
| Depth (D) | 3 | 73.77 | 1599.04 | **<0.0001** |  |
| S**×**D | 6 | 80.13 | 56.01 | **<0.0001** |  |
| W**×**D | 3 | 73.77 | 13.77 | **<0.0001** |  |
| S**×**W**×**D | 6 | 80.13 | 1.60 | 0.1584 |  |

**^†^**Significant effects (*p* < 0.05) are indicated in **bold**.

**
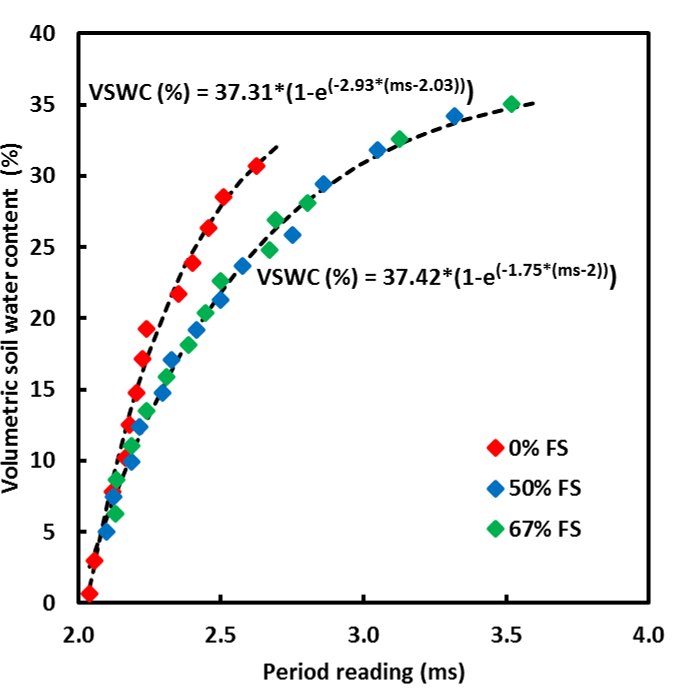
**

Supplementary Figure 1: Volumetric soil water content (VSWC; %) as a function of time-domain reflectometry (TDR) millisecond (ms) readings. TDR calibration best-fit curves (dashed lines) are plotted with data collected (symbols) on three different soil mixtures represented with three different colored symbols. Equations for the fitted curves are given. A single curve (equation) describes the relationship for both mixtures containing field soil (FS; 50% and 67% FS mix).

Supplementary Figure 2. Volumetric soil water content (VSWC; %) by depth for the three growth media tested, measured at five days after planting (5 DAP). Growth media treatments are 67% field soil (FS), 50% FS or 0% FS mixes, watered daily to 100% soil water holding capacity (Control). VSWC measurements were taken 24 h after the previous watering. Data are the means of 12 tubes (plants) ± 1 s.e.m. in each growth medium treatment. If not seen, the standard error is smaller than the symbol.

Supplementary Figure 3. Volumetric soil water content (VSWC; %) by depth for the three growth media tested, measured at different days after planting (DAP): panel A (35 DAP; top), and panel B (40 DAP; bottom). Growth media treatments are 67% field soil (FS), 50% FS or 0% FS mixes, watered daily to either 100% soil water holding capacity (SWHC; Control) or 75% SWHC (Stress). VSWC measurements were taken 24 h after the previous watering. Data represent the soil mix by watering treatment interaction least square mean values ± 1 s.e.m. Six replicates were used. If not seen, the standard error is smaller than the symbol.

Supplementary Figure 4. Volumetric soil water content by depth for the 67% field soil mix, measured at 6 h, 24 h, and 48 h after watering at 35 days after planting (35 DAP). Figure (A) represents tubes that were watered to 100% soil water holding capacity (SWHC; Control) whereas figure B represents the tubes that were watered to 75% SWHC (Stress). Data represent the least square mean values ± 1 s.e.m. in each water stress treatment over time. Six replicates were used. If not seen, the standard error is smaller than the symbol.
